# Sclavo's Serum in the Cure of Anthrax without Operation

**Published:** 1910-07-02

**Authors:** 


### Medicine.

### SCLAVO'S SERUM IN THE CURE OF ANTHRAX WITHOUT OPERATION.

Anthrax in the human being is fortunately uncommon in most parts of Great Britain, but there are certain districts in which it is liable to occur, notably in Bermondsey and in Bradford, where hides or wool or hair sometimes introduce the affection. Rare though the condition may be, its early recognition and its active treatment are of importance if life is to be saved. The commonest form of the affection is malignant pustule on some exposed part, particularly of the face, neck, hand, or arm. In the past it has been considered that the local pustule and the skin around it should be excised freely and as early as possible whilst the micro-organisms are still local to the one spot; for if the latter is left it is recognised that there is a great danger of a spread of the infection to the general circulation, in which case the termination is almost certain to be fatal.

Of recent years another form of treatment—namely, that of Sclavo's anti-anthrax serum—has come into vogue, and although it may be safest to excise the pustule as well as to use the serum, there have been not a few patients in whom successful treatment has resulted from the use of the serum alone. The possible nature of the lesion will be suggested, as a rule, by the patient's occupation, and then the diagnosis will be confirmed on bacteriological examination by the discovery of the characteristic large gram-positive micro-organisms.

The following case, as recorded by Dr. Armour in the *Liverpool Medico-Chirurgical Journal*,* was one in which it was not thought advisable to attempt excision of the affected area. Treatment was confined to the local application of antiseptics and to the subcutaneous injection of the serum. It is not claimed that the serum was entirely responsible for the satisfactory termination of the case, as the infection was not extremely virulent, judged from the constitutional disturbance; but it is certain that the injections did diminish the severity of the general symptoms, prevented aggravation of the local lesion, and hastened convalescence very considerably. W. H., aged fifty-four, was admitted to hospital on the evening of April 28, 1909. He gave a history of having helped in the discharge of a cargo of hides from Pernambuco on April 20, and said that he had not worn gloves when handling the hides. Six days afterwards he noticed a small pimple on his neck, and as this and the surrounding swelling got rapidly worse and he began to have difficulty with his breathing, he came to the hospital. It was found that there was a typical malignant pustule situated in the middle line of his neck, just below the cricoid cartilage; there was a small black slough about the size of a pea, surrounded by a ring of vesicles and much brawny œdema of the neck in the neighbourhood. The redness and swelling extended on either side as far as a perpendicular line dropped from the mastoid process, and the supraclavicular and suprasternal fossæ were largely obliterated. There was some dyspnœa and the face was slightly cyanosed, the temperature was 102, and the pulse-rate 96. Anthrax bacilli were found in the serum taken from the vesicles. It was decided, however, that excision of the affected tissue was not feasible on account of the underlying trachea and great vessels, and an application of pure carbolic acid to the pustule and warm 1 in 40 carbolic fomentations were ordered, and the injection of Sclavo's serum as soon as it could be obtained. On the following day the patient's condition was worse; he was very restless and at times delirious, his temperature was 103, the swelling had extended upwards in his neck nearly to the level of the angles of his lower jaw, he was unable to swallow even fluids, and his breathing was very difficult and accompanied by stridor. He appeared in imminent danger of suffocation from œdema glottidis. It was thought that tracheotomy would inevitably result in infection of the trachea and lungs on account of the position of the pustule, and the man was so restless that there was considerable doubt as to intubation being successful. Luckily, however, frequent steam inhalations relieved the breathing greatly. In the evening the serum arrived from London, and 20 c.c. were at once injected under the skin of the anterior abdominal wall. The patient had a much better night; he slept at intervals, and the breathing improved considerably; he was able to swallow quantities of fluid, and within twelve hours of the injection of the serum the temperature fell to 99 deg. It remained at this level for six hours and then rose to 103 deg. again. On April 30 two injections of 30 c.c. each were given, one in the morning and the other in the evening. On May 1 the temperature fell to 98.8 deg. in the morning, and did not rise above 100 deg. during the rest of the time the patient was in hospital. The dyspnœa and dysphagia disappeared the day after the third injection, and by May 3, when the serum was discontinued, the man was practically convalescent. The œdema of the neck had diminished so much that the natural hollows and prominences could be observed, and the pustule had become a drying black slough about the size of a florin, surrounded by a slightly reddened area; there were now no vesicles, nor was there much discharge. The slough did not separate for ten days, and when it did it was replaced by a smaller slough; this process was repeated more than once, and the final slough did not come away till June 21. By the end of June the neck was completely healed.

Sclavo obtains his anti-anthrax serum from the blood of an immunised ass. He found that this animal provides a stronger serum than sheep or goats. The ass is immunised most quickly by combining the inoculation with Pasteur's attenuated anthrax cultures with the injection of serum from an immune animal; the animal then receives a dose of virulent culture.

* January 1910.

